# Twin Least Square Support Vector Regression Model Based on Gauss-Laplace Mixed Noise Feature with Its Application in Wind Speed Prediction

**DOI:** 10.3390/e22101102

**Published:** 2020-09-29

**Authors:** Shiguang Zhang, Chao Liu, Wei Wang, Baofang Chang

**Affiliations:** 1 College of Computer and Information Engineering, Henan Normal University, Xinxiang 453007, China; zhangshiguang@htu.edu.cn (S.Z.); wangwei@htu.edu.cn (W.W.); changbaofang@htu.edu.cn (B.C.); 2 School of Computer Science and Technology, Tianjin University, Tianjin 300350, China; 3 Engineering Lab of Intelligence Business and Internet of Things, Xinxiang 453007, China

**Keywords:** Gauss-Laplace mixed noise, least squares support vector regression, twin hyperplanes, wind speed prediction

## Abstract

In this article, it was observed that the noise in some real-world applications, such as wind power forecasting and direction of the arrival estimation problem, does not satisfy the single noise distribution, including Gaussian distribution and Laplace distribution, but the mixed distribution. Therefore, combining the twin hyperplanes with the fast speed of Least Squares Support Vector Regression (LS-SVR), and then introducing the Gauss–Laplace mixed noise feature, a new regressor, called Gauss-Laplace Twin Least Squares Support Vector Regression (GL-TLSSVR), for the complex noise. Subsequently, we apply the augmented Lagrangian multiplier method to solve the proposed model. Finally, we apply the short-term wind speed data-set to the proposed model. The results of this experiment confirm the effectiveness of our proposed model.

## 1. Introduction

In recent years, the support vector machine (SVM) [1,2,3,4] have received widespread attention as a powerful method, because support vector machines have better generalization performance than other machine learning techniques. Thanks to good generalization capabilities, SVM technology is applied to various fields. For example, SVM has been applied to face detection [5], feature selection [6], function approximation [7], financial forecasting [8], and wind turbine system [9,10,11,12,13,14,15]. As for the support vector regression (SVR) model [16], it uses support vector machine technology to solve the regression estimation, there are many important methods, such as ε-support vector regression (ε-SVR) [17], ν-support vector regression (ν-SVR) [18] etc. In addition, based on some advantages of SVR, SVR has been successfully applied to Biology, medicine, environmental protection, information technology, engineering technology, and other fields [19,20,21,22,23,24].

In these SVR models, when solving regression problems, the noise of the training data is considered to be the single distribution. According to the Bayesian principle, first, the Gaussian noise with square loss is the best, secondly, the Beta noise with Beta loss is the best, finally, the Laplace noise with Laplace loss is the best [25,26]. However, in some practical applications, if data are collected in a multi-source environment, then the noise distribution is complex and unknown. Therefore, a single distribution cannot clearly describe the real noise [27,28]. In general, the mixed distribution has a good approximation ability for any continuous distribution. For some actual noises, prior knowledge is difficult to obtain. At this time, mixed noise can be well adapted to unknown or complex noise. In 2017, the research and application of a new wind speed hybrid forecasting system that is based on multi-objective optimization is proposed [27,29], the proposed hybrid model is integrated with three components, singular spectrum analysis, the firefly algorithm, and the BP neural network [30]; as compared with a single BP, the prediction effect of the hybrid prediction method is better, which shows that the prediction ability of the hybrid method is stronger. In addition, accurate prediction of wind speed is a key task for the development and utilization of wind energy, when compared with other related methods, the proposed hybrid method has satisfactory performance in terms of accuracy and stability [31]. In this literature [32], two new nonlinear regression models for single-task and multi-task problems are developed, in which the noise is composed of Gaussian mixture. When compared to some other models, the proposed model becomes a robust nonlinear regression model with strong adaptation.

However, the main disadvantage of SVR is the high cost of learning. In order to improve the calculation speed of SVR, based on twin support vector machine (TSVM) [33], Peng [34,35,36] proposed twin support vector regression (TSVR). Unlike SVR, TSVR generates two non-parallel upper and lower bound functions by solving a pair of smaller quadratic programming problems (QPPs). In theory, TSVR reduces the computational cost compared to standard SVR. Zhao et al. [37] extended the concept of twin hyperplanes, and combined the advantages of least squares support vector regression (LSSVR) to generate the estimated regressor, called Twin Least Squares Support Vector Regression (TLSSVR). By observing the model of Peng [34], Khemchandani et al. [38] believed that only the principle of empirical risk minimization was considered in TSVR. To overcome these difficulties, Shao et al. [39] proposed another twin regression model, called ε-TSVR, which considers the principle of structural risk minimization. Later, Rastogi et al. [40] extended ε-TSVR and proposed ν-TSVR, which can automatically optimize parameters ε1 and ε2 based on sample data. By using the pinball loss function, Xu et al. [41] further developed an asymmetric ν-twin support vector regression, called Asy-ν-TSVR, which can effectively reduce noise interference and improve the generalization performance. Therefore, extensive research has been conducted on the twin-type SVR. In all of these twin-type SVR models, the distribution of training data is not considered in solving regression problems. This means that, regardless of whether the samples are important or not, all of the samples play the same role in the constraint function, so it will cause regression performance to decline. Depending on the importance of the data, given different samples, the penalty is more reasonable. For this reason, various methods [42,43,44,45,46] have been developed in order to study this shortcoming. For example, Xu et al. [44] proposed using the local information present on the sample based on K-nearest neighbor weighted twin support vector regression to improve the prediction accuracy. By clustering based on the similarity of training data, Parastalooi et al. [45] proposed an improved twin support vector regression. Ye [46] proposed an effective weighted Lagrangian ε-twin support vector regression (WL-ε-TSVR) with quadratic loss function, in which the weight matrix D was introduced in order to reduce the outlier pair to a certain extent Regression of the influence of variables, so as to impose different penalties on samples.

Traditionally, the upper and lower regression of the twin SVR is obtained by approximate dual solutions. However, Chapelle [47] observed that, by comparing the approximate efficiency of SVR in the primal space and the dual space, the approximate dual solution may not produce a good primal approximate solution. Some related work is directly solved in the primal space [48,49,50,51]. For example, inspired by the twin SVR and Newton methods, Balasundaram et al. [49] proposed a new unconstrained Lagrangian TSVR (ULTSVR) to solve a pair of unconstrained minimization problems, thereby increasing the calculation speed. Gupta [50] and Balasundaram [51] use the generalized derivative method to obtain QPPs. Although their work is efficient and fast, they only consider empirical risk minimization and do not consider structural risks.

Inspired by the above research, we try to study the characteristics of the complex or unknown noise distribution of the Gauss–Laplace mixed noise twin least squares support vector regression (GL-TLSSVR) model. In this article, for the solution to the regression task, the augmented Lagrange multiplier method (ALM) algorithm is used in our experiments, it can help us better to find the optimal solution.

**Figure 1:**
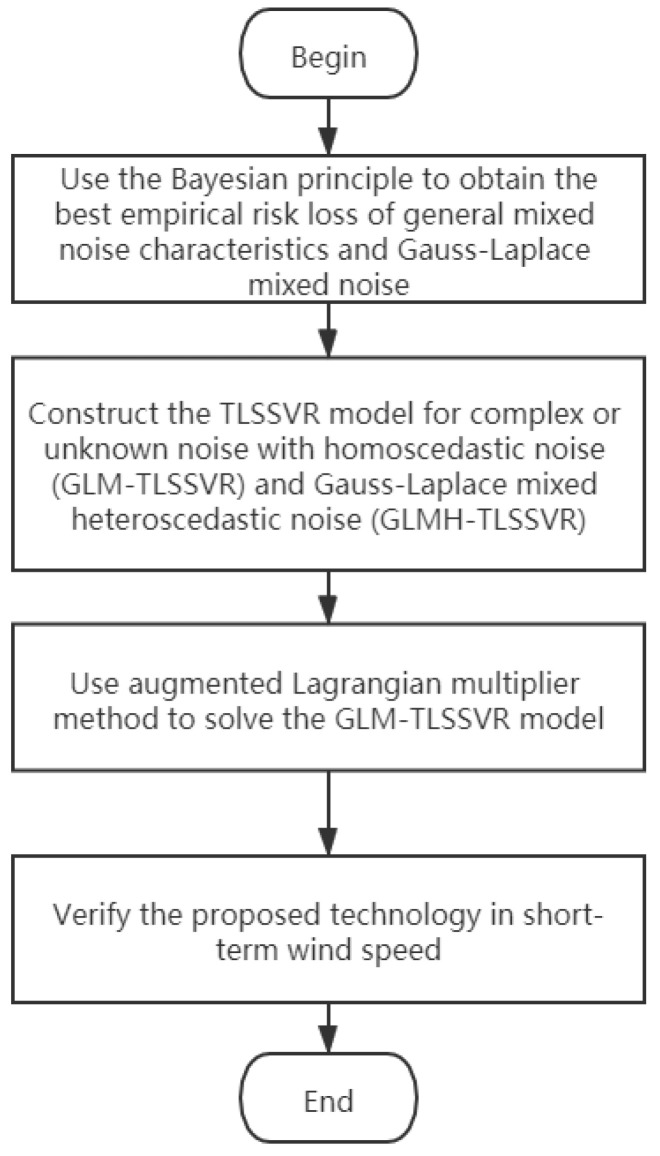
The whole methodology process of this article.

This work mainly provides four contributions, we describe the whole methodology in the flowchart, as shown in Figure 1.

## 2. Related Work

In this section, the data-set is represented by $D_{N}=\left\{\left(A_{i},y_{i}\right)\right\},i=1,2,\ldots ,N$, where $A_{i}\in R^{n}$, $y_{i}\in R\left(i=1,2,\ldots ,N\right)$ is the training samples.

According to the Bayesian principle, we can derive the optimal empirical risk loss of the mixed noise characteristics [52]. The best empirical risk loss for this mixed noise distribution is shown below

(1) $$l\left(\zeta \right)=\lambda_{1}\cdot l_{1}\left(\zeta \right)+\lambda_{2}\cdot l_{2}\left(\zeta \right).$$

where $l_{1}\left(\zeta \right)>0,l_{2}\left(\zeta \right)>0$ are the convex empirical risk loss of the above two noise characteristics. $\lambda_{1},\lambda_{2}\geq 0$ are weight factors, and $\lambda_{1}+\lambda_{2}=1$.

**Figure 2:**
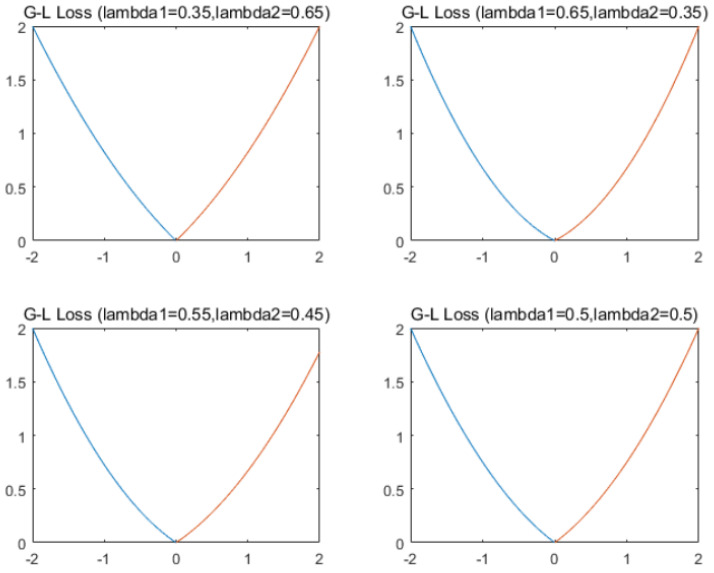
G-L empirical risk loss for different parameters.

Figure 2 shows the G-L empirical risk loss for different parameters.

## 3. TLSSVR Model of G-L Mixed Noise Characteristics

For the linear model, we want to find a linear regression function $f\left(A\right)=\varpi^{T}\cdot A+b$. When dealing with some nonlinear problems, some specific methods are given ([53]): the input vector $A_{i}\in R^{n}$ is mapped by a non-linear mapping Φ: $R^{n}\to H$ (take a prior distribution) to the high dimensional feature space *H* (*H* is Hilbert space), induced by the nonlinear kernel function $K\left(A_{i},A_{j}\right)=\left(\Phi \left(A_{i}\right)\cdot \Phi \left(A_{j}\right)\right)$(i,j=1,2,⋯,N), $\left(\Phi \left(A_{i}\right)\cdot \Phi \left(A_{j}\right)\right)$ is the inner product in *H*.

The twin least squares support vector regression model with mixed noise characteristics (M-TLSSVR) is proposed. The primal problem with model M-TLSSVR is shown below

(2) $$\begin{array}{c} Min\{{g_{P_{M}}}_{{{}_{-}}_{TLSSVR}}=\frac{1}{2}\omega_{1}^{T}\cdot \omega_{1}+\frac{C_{1}}{N}\cdot \left[\lambda_{1}\cdot \sum_{i=1}^{N}(l_{1}\left(\xi_{i}\right))+\lambda_{2}\cdot \sum_{i=1}^{N}\left(l_{2}\left(\xi_{i}\right)\right)\right]\}\\ s.t.\;\;\;\;\;\;y_{i}=\omega_{1}^{T}\cdot \phi \left(A_{i}\right)+b_{1}-\xi_{i} \end{array}$$

(3) $$\begin{array}{c} Min\{{g_{P_{M}}}_{{{}_{-}}_{TLSSVR}}=\frac{1}{2}\omega_{2}^{T}\cdot \omega_{2}+\frac{C_{2}}{N}\cdot \left[\lambda_{3}\cdot \sum_{i=1}^{N}(l_{1}\left(\xi_{i}^{*}\right))+\lambda_{4}\cdot \sum_{i=1}^{N}\left(l_{2}\left(\xi_{i}^{*}\right)\right)\right]\}\\ s.t.\;\;\;\;\;\;y_{i}=\omega_{2}^{T}\cdot \phi \left(A_{i}\right)+b_{2}+\xi_{i}^{*} \end{array}$$

where $\varpi_{1}$, $\varpi_{2}$ denotes the weight vector and $b_{1}$, $b_{2}$ is the bias term, Φ(A) is the nonlinear mapping that transfers the input vector to a higher-dimensional feature space. $\xi_{i}$, $\xi_{i}^{*}$ are random slack variable at time *i*. $l_{1}\left(\xi_{i}\right),l_{1}\left(\xi_{i}^{*}\right),l_{2}\left(\xi_{i}\right),l_{2}\left(\xi_{i}^{*}\right)>0\left(i=1,2,\cdots ,N\right)$ be general convex empirical risk loss values for noise characteristic in the sample point $\left(A_{i},y_{i}\right)\in D_{N}$ ((i,j=1,2,⋯,N)). $C_{1}>0$, $C_{2}>0$ be the penalty parameter, weight factor $\lambda_{1},\lambda_{2},\lambda_{3},\lambda_{4}\geq 0$, and $\lambda_{1}+\lambda_{2}=1$, $\lambda_{3}+\lambda_{4}=1$.

According to the literature [28], the mixed noise model is distributed by multiple noises, and its performance is better than the single noise model. In this section, Gauss–Laplace mixed homoscedastic and heteroscedastic noise distributions are used to describe complex noise characteristics.

### 3.1. TLSSVR Model of G-L Mixed Homoscedastic Noise Characteristics

According to Bayesian principle, it concludes that the empirical risk loss of the homoscedastic Gaussian noise of the lower bound function is $l_{1}\left(\xi \right)=\frac{1}{2\sigma^{2}}\cdot \xi^{2}$, the Laplace noise is $l_{2}\left(\xi \right)=\left|\xi \right|$. Adopting G-L mixed homoscedastic noise distribution to fit complicated noise-characteristic, by Equation (1), the empirical risk loss about G-L mixed homoscedastic noise is $l\left(\xi \right)=\frac{\lambda_{1}}{2\sigma^{2}}\cdot \xi^{2}+\lambda_{2}\cdot \left|\xi \right|$. The lower bound function of the G-L mixed homoscedastic noise characteristic TLSSVR model (GLM-TLSSVR) is proposed, the primal problem of the lower bound function is depicted as

(4) $$\begin{array}{c} Min\{{g_{P_{GLM}}}_{{{}_{-}}_{TLSSVR}}=\frac{1}{2}\omega_{1}^{T}\cdot \omega_{1}+\frac{C_{1}}{N}\cdot \left(\frac{\lambda_{1}}{2\sigma^{2}}\cdot \sum_{i=1}^{N}{\xi_{i}}^{2}+\lambda_{2}\cdot \sum_{i=1}^{N}\left|\xi_{i}\right|\right)\}\\ s.t.\;\;\;\;\;\;y_{i}=\omega_{1}^{T}\cdot \phi \left(A_{i}\right)+b_{1}-\xi_{i} \end{array}$$

Similarly, we can get that the primal problem of the upper bound function of the model GLM-TLSSVR is

(5) $$\begin{array}{c} Min\{{g_{P_{GLM}}}_{{{}_{-}}_{TLSSVR}}=\frac{1}{2}\omega_{2}^{T}\cdot \omega_{2}+\frac{C_{2}}{N}\cdot \left(\frac{\lambda_{3}}{2\sigma^{{*}^{2}}}\cdot \sum_{i=1}^{N}{\xi_{i}}^{{*}^{2}}+\lambda_{4}\cdot \sum_{i=1}^{N}\left|\xi_{i}^{*}\right|\right)\}\\ s.t.\;\;\;\;\;\;y_{i}=\omega_{2}^{T}\cdot \phi \left(A_{i}\right)+b_{2}+\xi_{i}^{*} \end{array}$$

Where $\xi_{i}$ and $\xi_{i}^{*}$ are the random noise and slack variables at time *i*. parameter vector $\omega_{1},\omega_{2}\in R^{n}$, $\sigma^{2},\sigma^{{*}^{2}}$ are homoscedastic, $C_{1},C_{2}>0$ are a penalty parameter, and the weight factors are $\lambda_{1},\lambda_{2},\lambda_{3},\lambda_{4}\geq 0$, $\lambda_{1}+\lambda_{2}=1,\lambda_{3}+\lambda_{4}=1$.

**Proposition** **1.** *The solution of primal problem (4), (5) of GLM-TLSSVR about $\varpi_{1}$, $\varpi_{2}$ exist and are unique.*

**Theorem** **1.** *The dual problem of primal problem (4) of GLM-TLSSVR is*

(6) $$\begin{array}{c} Max\{g_{D_{GLM-TLSSVR}}=-\frac{1}{2}\sum_{i=1}^{N}\sum_{j=1}^{N}\left(\alpha_{i}+\beta_{i}\right)\cdot \left(\alpha_{j}+\beta_{j}\right)\cdot K\left(A_{i},A_{j}\right)-\\ \frac{C_{1}\sigma^{2}}{N}\cdot \frac{\lambda_{2}^{2}}{\lambda_{1}}\cdot \sum_{i=1}^{N}\frac{\alpha_{i}}{\beta_{i}}-\frac{N\sigma^{2}}{2C_{1}\lambda_{1}}\sum_{i=1}^{N}\alpha_{i}^{2}\}\\ s.t.\;\;\;\;\;\;\sum_{i=1}^{N}(\alpha_{i}+\beta_{i})=0 \end{array}$$

*The dual Problem of primal problem (5) of GLM-TLSSVR is*

(7) $$\begin{array}{c} Max\{g_{D_{GLM-TLSSVR}}=-\frac{1}{2}\sum_{i=1}^{N}\sum_{j=1}^{N}\left(\alpha_{i}^{*}+\beta_{i}^{*}\right)\cdot \left(\alpha_{j}^{*}+\beta_{j}^{*}\right)\cdot K\left(A_{i},A_{j}\right)-\\ \frac{C_{2}\sigma^{{*}^{2}}}{N}\cdot \frac{\lambda_{4}^{2}}{\lambda_{3}}\cdot \sum_{i=1}^{N}\frac{\alpha_{i}^{*}}{\beta_{i}^{*}}-\frac{N\sigma^{{*}^{2}}}{2C_{2}\lambda_{3}}\cdot \sum_{i=1}^{N}\alpha_{i}^{{*}^{2}}\}\\ s.t.\;\;\;\;\;\;\sum_{i=1}^{N}(\alpha_{i}^{*}+\beta_{i}^{*})=0 \end{array}$$

where parameter vector $\omega_{1},\omega_{2}\in R^{n}$, $\sigma^{2},\sigma^{{*}^{2}}$ are homoscedastic, $C_{1},C_{2}>0$ are a penalty parameter, and the weight factors are $\lambda_{1},\lambda_{2},\lambda_{3},\lambda_{4}\geq 0$, $\lambda_{1}+\lambda_{2}=1,\lambda_{3}+\lambda_{4}=1$, $\alpha_{i},\alpha_{j},\alpha_{i}^{*},\alpha_{j}^{*}$ are the Lagrange multiplier.

**Proof.**  On the lower bound function of the GLM-TLSSVR model, for any vector *u*, If we set $u_{\pm }\geq 0$ to $u=u_{+}-u_{-}$, then $\min\left|u\right|=\min\left\{u_{+}+u_{-}\right\}$ will be established [54]. Therefore, by setting $\xi_{i}=p_{i}-r_{i}$, $r_{i},p_{i}\geq 0$, the primal problem of the lower bound function of GLM-TLSSVR is simplified, as follows

(8) $$\begin{array}{c} Min\{{g_{P_{GLM}}}_{{{}_{-}}_{TLSSVR}}=\frac{1}{2}\omega_{1}^{T}\cdot \omega_{1}+\frac{C_{1}}{N}\cdot \left[\frac{\lambda_{1}}{2\sigma^{2}}\cdot \sum_{i=1}^{N}{\xi_{i}}^{2}+\lambda_{2}\cdot \sum_{i=1}^{N}\left(r_{i}+p_{i}\right)\right]\}\\ s.t.\;\;\;\;\;\;y_{i}-\left(\omega_{1}^{T}\phi \left(A_{i}\right)+b_{1}\right)-r_{i}+p_{i}=0\\ \;\;\;\;\;\;y_{i}=\omega_{1}^{T}\phi \left(A_{i}\right)+b_{1}-\xi_{i}\\ \;\;\;\;\;\;r_{i},p_{i}\geq 0\left(i=1,\ldots ,N\right) \end{array}$$

We introduce the Lagrange function and KKT(Karush–Kuhn–Tucker) condition [55]. □

We get the solution of the lower bound function

$$\omega_{1i}=\sum_{i=1}^{N}\left(\alpha_{i}+\beta_{i}\right)\cdot \phi \left(A_{i}\right),$$

$$b_{1}=\sum_{i=1}^{N}[y_{i}-\sum_{j=1}^{N}\left(\alpha_{i}+\beta_{i}\right)\cdot K\left(A_{i},A_{j}\right)-\frac{1}{\lambda_{1}}\cdot \frac{N\cdot \sigma^{2}\cdot \alpha_{i}}{C_{1}}].$$

Thus, the lower function of model TLSSVR with Gauss–Laplace mixture homoscedastic noise characteristic (GLM-TLSSVR) can be written as

$$f_{1}\left(A\right)=\omega_{1}^{T}\cdot \phi \left(A\right)+b_{1}=\sum_{i=1}^{N}\left(\alpha_{i}+\beta_{i}\right)K\left(A_{i},A\right)+b_{1}$$

**Theorem** **2.** *The primal problem of the upper bound function of GLM-TLSSVR is simplified, as follows*

(9) $$\begin{array}{c} Min\{{g_{P_{GLM}}}_{{{}_{-}}_{TLSSVR}}=\frac{1}{2}\omega_{2}^{T}\cdot \omega_{2}+\frac{C_{2}}{N}\cdot \left(\frac{\lambda_{3}}{2\sigma^{{*}^{2}}}\cdot \sum_{i=1}^{N}{\xi_{i}}^{{*}^{2}}+\lambda_{4}\cdot \sum_{i=1}^{N}\left(r_{i}+p_{i}\right)\right)\}\\ s.t.\;\;\;\;\;\;y_{i}-\left(\omega_{2}^{T}\phi \left(A_{i}\right)+b_{2}\right)+r_{i}-p_{i}=0\\ \;\;\;\;\;\;y_{i}=\omega_{2}^{T}\phi \left(A_{i}\right)+b_{2}+\xi_{i}^{*}\\ \;\;\;\;\;\;r_{i},p_{i}\geq 0\left(i=1,\ldots ,N\right) \end{array}$$

*Similarly, we introduce the Lagrange function and KKT conditions again.*

We obtain the solution of the upper bound function

$$\omega_{2i}=\sum_{i=1}^{N}\left(\alpha_{i}^{*}+\beta_{i}^{*}\right)\cdot \phi \left(A_{i}\right),$$

$$b_{2}=\sum_{i=1}^{N}[y_{i}-\sum_{j=1}^{N}\left(\alpha_{i}^{*}+\beta_{i}^{*}\right)\cdot K\left(A_{i},A_{j}\right)-\frac{1}{\lambda_{3}}\cdot \frac{N\cdot \sigma^{{*}^{2}}\cdot \alpha^{*}}{C_{2}}].$$

Thus, the upper function of model TLSSVR with Gauss–Laplace mixture homoscedastic noise characteristic (GLM-TLSSVR) can be written as

$$f_{2}\left(A\right)=\omega_{2}^{T}\cdot \phi \left(A\right)+b_{2}=\sum_{i=1}^{N}\left(\alpha_{i}^{*}+\beta_{i}^{*}\right)K\left(A_{i},A\right)+b_{2}$$

At last, the estimated regressor of GLM-TLSSVR is written, as follows

$$f\left(A\right)=\frac{\omega_{1}^{T}+\omega_{2}^{T}}{2}\cdot \phi \left(A\right)+\frac{b_{1}+b_{2}}{2}=\sum_{i=1}^{N}\frac{\alpha_{i}+\beta_{i}+\alpha_{i}^{*}+\beta_{i}^{*}}{2}\cdot K\left(A_{i},A\right)+\frac{b_{1}+b_{2}}{2}$$

where, parameter vector $\omega_{1},\omega_{2}\in R^{n},\phi :R^{n}\to H,\left(\phi \left(A_{i}\right)\cdot \phi \left(A_{j}\right)\right)$ is the inner product of *H*, $K\left(A_{i},A_{j}\right)=\left(\phi \left(A_{i}\right)\cdot \phi \left(A_{j}\right)\right)$ is the kernel function.

### 3.2. TLSSVR Model of G-L Mixed Heteroscedastic Noise Characteristics

If the noise is Gaussian noise with zero mean and variance of heteroscedasticity, and these variance are $\sigma_{i}^{2},{\left(\sigma_{i}^{*}\right)}^{2}$, where $\sigma_{i}\neq \sigma_{j}$, $\sigma_{i}^{*}\neq \sigma_{j}^{*}$, i≠j(i,j=1,⋯,N). Similarly, by Equation (1), the empirical risk loss is $l\left(\xi_{i}\right)=\frac{\lambda_{1}}{2\sigma_{i}^{2}}\cdot \xi_{i}^{2}+\lambda_{2}\cdot \left|\xi_{i}\right|,\left(i=1,\ldots ,N\right)$. A TLSSVR model for Gauss–Laplace mixed heteroscedastic noise characteristics is established, and we named it GLMH-TLSSVR. A pair of optimization problems of GLMH-TLSSVR can be depicted:

(10) $$\begin{array}{c} Min\{{g_{P_{GLM}}}_{{{}_{-}}_{TLSSVR}}=\frac{1}{2}\omega_{1}^{T}\cdot \omega_{1}+\frac{C_{1}}{N}\cdot \left(\frac{\lambda_{1}}{2\sigma_{i}^{2}}\cdot \sum_{i=1}^{N}{\xi_{i}}^{2}+\lambda_{2}\cdot \sum_{i=1}^{N}\left|\xi_{i}\right|\right)\}\\ s.t.\;\;\;\;\;\;y_{i}=\omega_{1}^{T}\cdot \phi \left(A_{i}\right)+b_{1}-\xi_{i} \end{array}$$

(11) $$\begin{array}{c} Min\{{g_{P_{GLM}}}_{{{}_{-}}_{TLSSVR}}=\frac{1}{2}\omega_{2}^{T}\cdot \omega_{2}+\frac{C_{2}}{N}\cdot \left(\frac{\lambda_{3}}{2\sigma_{i}^{{*}^{2}}}\cdot \sum_{i=1}^{N}{\xi_{i}}^{{*}^{2}}+\lambda_{4}\cdot \sum_{i=1}^{N}\left|\xi_{i}^{*}\right|\right)\}\\ s.t.\;\;\;\;\;\;y_{i}=\omega_{2}^{T}\cdot \phi \left(A_{i}\right)+b_{2}+\xi_{i}^{*} \end{array}$$

where $\xi_{i}$ and $\xi_{i}^{*}$ are random noise and slack variables at time *i*. These heteroscedastic variables are $\sigma_{i}^{2}.{\left(\sigma_{i}^{*}\right)}^{2}\left(i=1,2,\cdots ,N\right)$, $C_{1},C_{2}>0$ are the penalty parameter, weight factor $\lambda_{1},\lambda_{2},\lambda_{3},\lambda_{4}\geq 0$, and $\lambda_{1}+\lambda_{2}=1,\lambda_{3}+\lambda_{4}=1$.

**Proposition** **2.** *The solution of primal problem (10), (11) of GLMH-TLSSVR about $\omega_{1},\omega_{2}$ exist and are unique.*

**Theorem** **3.** *The dual problem of GLMH-TLSSVR in primal problem (10), (11) be*

(12) $$\begin{array}{c} Max\{g_{D_{GLM-TLSSVR}}=-\frac{1}{2}\sum_{i=1}^{N}\sum_{j=1}^{N}\left(\alpha_{i}+\beta_{i}\right)\cdot \left(\alpha_{j}+\beta_{j}\right)\cdot K\left(A_{i},A_{j}\right)-\\ \frac{C_{1}\sigma_{i}^{2}}{N}\cdot \frac{\lambda_{2}^{2}}{\lambda_{1}}\cdot \sum_{i=1}^{N}\frac{\alpha_{i}}{\beta_{i}}-\frac{N\sigma_{i}^{2}}{2C_{1}\lambda_{1}}\sum_{i=1}^{N}\alpha_{i}^{2}\}\\ s.t.\;\;\;\;\;\;\sum_{i=1}^{N}(\alpha_{i}+\beta_{i})=0 \end{array}$$

(13) $$\begin{array}{c} Max\{g_{D_{GLM-TLSSVR}}=-\frac{1}{2}\sum_{i=1}^{N}\sum_{j=1}^{N}\left(\alpha_{i}^{*}+\beta_{i}^{*}\right)\cdot \left(\alpha_{j}^{*}+\beta_{j}^{*}\right)\cdot K\left(A_{i},A_{j}\right)-\\ \frac{C_{2}\sigma_{i}^{{*}^{2}}}{N}\cdot \frac{\lambda_{4}^{2}}{\lambda_{3}}\cdot \sum_{i=1}^{N}\frac{\alpha_{i}^{*}}{\beta_{i}^{*}}-\frac{N\sigma_{i}^{{*}^{2}}}{2C_{2}\lambda_{3}}\cdot \sum_{i=1}^{N}\alpha_{i}^{{*}^{2}}\}\\ s.t.\sum_{i=1}^{N}(\alpha_{i}^{*}+\beta_{i}^{*})=0 \end{array}$$

**Proof.**  Similar to Theorems 1 and 2, an appendix to the proof of Theorem 3. □

We can obtain the solution of the lower bound function

$$\omega_{1i}=\sum_{i=1}^{N}\left(\alpha_{i}+\beta_{i}\right)\cdot \phi \left(A_{i}\right),$$

$$b_{1}=\sum_{i=1}^{N}[y_{i}-\sum_{j=1}^{N}\left(\alpha_{i}+\beta_{i}\right)\cdot K\left(A_{i},A_{j}\right)-\frac{1}{\lambda_{1}}\cdot \frac{N\cdot \sigma_{i}^{2}\cdot \alpha_{i}}{C_{1}}].$$

Thus, the lower function of model TLSSVR with Gauss–Laplace mixture heteroscedastic noise characteristics (GLMH-TLSSVR) can be written as

$$f_{1}\left(A\right)=\omega_{1}^{T}\cdot \phi \left(A\right)+b_{1}=\sum_{i=1}^{N}\left(\alpha_{i}+\beta_{i}\right)K\left(A_{i},A\right)+b_{1}$$

We also get the solution of the upper bound function

$$\omega_{2i}=\sum_{i=1}^{N}\left(\alpha_{i}^{*}+\beta_{i}^{*}\right)\cdot \phi \left(A_{i}\right),b_{2}=\sum_{i=1}^{N}[y_{i}-\sum_{j=1}^{N}\left(\alpha_{i}^{*}+\beta_{i}^{*}\right)\cdot K\left(A_{i},A_{j}\right)-\frac{1}{\lambda_{3}}\cdot \frac{N\cdot \sigma_{i}^{{*}^{2}}\cdot \alpha^{*}}{C_{2}}].$$

The upper function of model TLSSVR with Gauss–Laplace mixture heteroscedastic noise characteristics (GLMH-TLSSVR) can be written as

$$f_{2}\left(A\right)=\omega_{2}^{T}\cdot \phi \left(A\right)+b_{2}=\sum_{i=1}^{N}\left(\alpha_{i}^{*}+\beta_{i}^{*}\right)K\left(A_{i},A\right)+b_{2}$$

At last, the estimated regressor of GLMH-TLSSVR is written, as follows

$$f\left(A\right)=\frac{\omega_{1}^{T}+\omega_{2}^{T}}{2}\cdot \phi \left(A\right)+\frac{b_{1}+b_{2}}{2}=\sum_{i=1}^{N}\frac{\alpha_{i}+\beta_{i}+\alpha_{i}^{*}+\beta_{i}^{*}}{2}\cdot K\left(A_{i},A\right)+\frac{b_{1}+b_{2}}{2}$$

If this noise characteristic is Gaussian with the homoscedasticity, we can use Theorem 3 in order to derive Theorem 1 and Theorem 2.

## 4. ALM Method Analysis

In this section, we apply the augmented Lagrange multiplier method (ALM) [56] to solve the duality problems in Equations (6) and (7) by applying gradient descent or Newton’s method to equality-constrained sequences. By eliminating the equality constraints, any equality constraints can be reduced to the equivalent unconstrained problem [57,58]. When we deal with some large-scale data sets, some rapid optimizations can combine these techniques with the proposed model. For example, the sequential minimum optimization (SMO) algorithm [59] and stochastic gradient appropriate (SDG) algorithm [60].

From Theorems 1–3, we can find that this ALM method can help us to effectively identify the GLM-TLSSVR and GLMH-TLSSVR models. In this section, the lower bound function and upper bound function of the GLM-TLSSVR model can be solved by the ALM method. Similarly, the lower bound function and upper bound function of the GLMH-TLSSVR model can also be solved by the ALM method. The specific algorithm steps are as follows

(1) Set data-set be $D_{N}=\left\{\left(A_{1},y_{1}\right),\left(A_{2},y_{2}\right),\ldots ,\left(A_{L},y_{N}\right)\right\}$, where $A_{i}\in R^{n}$, $y_{i}\in R$, i=1,…,N.

(2) Select the appropriate kernel function through the 10-fold cross-validation strategy and obtain the appropriate parameters $C_{1},C_{2},\lambda_{1},\lambda_{2},\lambda_{3},\lambda_{4}$ of the lower and upper bound function of the model GLM-TLSSVR.

(3) When the optimization problem is solved in Equations (6) and (7), we can obtain the optimal solution $\alpha =\left(\alpha_{1},\ldots ,\alpha_{N}\right),\alpha^{*}=\left(\alpha_{1}^{*},\ldots ,\alpha_{N}^{*}\right),\beta =\left(\beta_{1},\ldots ,\beta_{N}\right),\beta^{*}=\left(\beta_{1}^{*},\ldots ,\beta_{N}^{*}\right)$.

(4) The decision function is established, as shown below

$$f\left(A\right)=\omega^{T}\cdot \phi \left(A\right)+b=\sum_{i=1}^{N}\frac{\alpha_{i}+\alpha_{i}^{*}+\beta_{i}+\beta_{i}^{*}}{2}\cdot K\left(A_{i},A\right)+b$$

## 5. Experiments and Discussion

In the section, to check the performance of the proposed model GLM-TLSSVR, we compared it with ν-SVR, LS-SVR, and TSVR on actual data-set from Heilongjiang, China. This part mainly includes three contents: G-L mixed noise characteristics of wind speed in Section 5.1; the criteria for algorithm evaluation in Section 5.2; and, application on predicting the short-term wind speed in Section 5.3.

### 5.1. G-L Mixed Noise Characteristics of Wind Speed

What we collected consists of one-year wind speed data-set from Heilongjiang Province, China. These data record the wind speed value every 10 min in order to better analyze the characteristics of mixed noise in the wind speed forecast error. In the above wind speed data, we found that some noise is a mixture of Gauss–Laplace. Some of the researchers have found that turbulence is the main cause of the uncertainty of strong random fluctuations in wind speed. From the perspective of wind energy, the most significant feature of wind resources is its variability.

**Figure 3:**
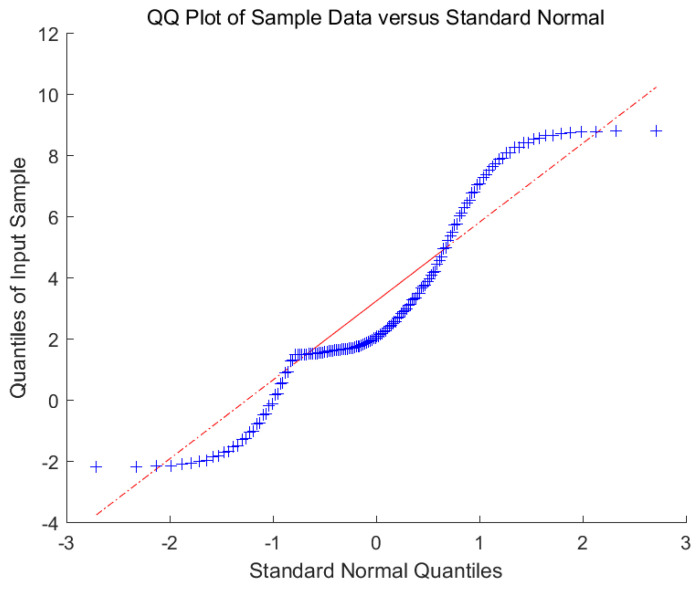
The wind speed forecast error distribution with mixed Gauss-Laplace noise. (This red line is used as a reference. It is determined by the quarter point and the third quarter point. These two points just determine the line in the QQ plot. These blue distribution points are the error between the actual value of wind speed and the predicted value of wind speed.).

We adopted the persistence method, which is often used to study the distribution of wind speed forecast errors, in order to analyze the wind speed data set of a one-month time series [54]. This experiment shows that the error variable ξ does not satisfy a single noise distribution, but approximately obeys the Gauss–Laplace mixed noise distribution, and the PDF of ξ is $P\left(\xi \right)=\frac{1}{2}e^{-|\xi |}\cdot \frac{1}{2\sigma^{2}}\xi^{2}$, we show the forecast error of Gauss–Laplace mixed wind speed distribution in Figure 3. It is found that this is a regression learning task about mixed noise.

### 5.2. The Criteria for Algorithm Evaluation

We specified evaluation criteria before introducing the experimental results in order to compare the performance of various models. The evaluation criteria are, as follows: the mean absolute error (MAE), the root mean square error (RMSE), sum of squared regression (SSR), sum of squared deviation of testing (SST), sum of squared error of testing (SSE), and teTime are used to evaluate the predictive performance of models ν-SVR, LS-SVR, TSVR, and GLM-TLSSVR. The five criteria are defined, as follows [34,37].

In Table 1, *L* is the number of testing samples, $y_{i}$ is the ith the real value, $y_{i}^{*}$ represents the predicted value, and $\bar{y}$ is the mean of the testing data-set. teTime(in seconds) represents the testing time of constructing a regressor.

### 5.3. Application on Predicting the Short-Term Wind Speed

In the section, we confirmed the feasibility and effectiveness of the proposed model GLM-TLSSVR on the short-term wind speed data set of Heilongjiang Province, China. The source of the wind speed data set is a related wind farm under the Meteorological Bureau of Heilongjiang Province, and a lightning imager measures the wind speed. This wind speed data set has been recorded for more than a year, and the average wind speed is recorded every 10 min. In general, we collected a total of 62,466 samples, which have four attributes, namely variance, mean, maximum, and minimum. We use 1440 uninterrupted data samples (from 1 to 1440, the time span is 10 days) as training samples. We also use 720 uninterrupted data samples (from 1441 to 2160, the time span is five days), and 80 consecutive data as the testing samples. As for the original sequence, we need to transform it into a multiple regression task by using mode ***$\vec{X_{i}}$ = $\left(X_{i-11},X_{i-10},\cdots ,X_{i-1},X_{i}\right)$*** as an input vector to predict $X_{i+step}$, where the vector orders of wind speed is determined by the chaotic operator network method. Where $X_{j}$ is the real value of wind speed at time j(j=i−11,i−10,⋯,i). In the experiments, we try step = 1, 3, and 5. In other words, we predicted the wind speed of every point $X_{i}$ after 10, 30, and 50 min, respectively.

These four models (ν-SVR, LS-SVR, TSVR, and GLM-TLSSVR) have been implemented in Python 3.7 on Windows 10 running on a PC with system configuration Intel i7 processor (3.19 GHz) with 8 GB of RAM. The initial parameters be $C_{1},C_{2}\in 2^{i}|i=-9,-8,\ldots ,10$, $\lambda_{1},\lambda_{2},\lambda_{3},\lambda_{4}\in \left[0,1\right]$. $C_{1},C_{2},\lambda_{1},\lambda_{2},\lambda_{3},\lambda_{4}$ are some tuned parameters by virtue of the 10-fold cross validation technique, where the cross validation technique is explained in detail in [61,62]. This technique can help us to find the optimal parameters. In this article, in order to reduce the computational burden of the GLM-TLSSVR model, the parameter assignments are, as follows: $C_{1}=C_{2}$, $\lambda_{1}=\lambda_{2}=\frac{1}{2},\lambda_{3}=\lambda_{4}=\frac{1}{2}$. As for the choice of kernel function, many experiments show that polynomial kernel function and Gaussian kernel function have good performance. In this experiment, we apply Gaussian kernel functions and polynomial kernel function to these four models (ν-SVR, LS-SVR, TSVR, and GLM-TLSSVR), as below [63].

$$K\left(X_{i},X_{j}\right)={\left(\left(X_{i},X_{j}\right)+1\right)}^{d},$$

$$K\left(X_{i},X_{j}\right)=e^{-\frac{∥X_{i}-X_{j}{∥}^{2}}{\sigma^{2}}},$$

where *d* is a positive integer, and σ is positive.

The dual problem of ν-SVR, LS-SVR, and TSVR are as follows.

ν-SVR: the authors of [18,61] define the dual problem of ν-SVR, as

(14) $$Max\{g_{D_{v-SVR}}=-\frac{1}{2}\sum_{i\in RSV}\sum_{j\in RSV}\left(\alpha_{i}^{*}-\alpha_{i}\right)\left(\alpha_{j}^{*}-\alpha_{j}\right)\cdot K\left(A_{i},A_{j}\right)\sum_{i\in RSV}\left(\alpha_{i}^{*}-\alpha_{i}\right)\cdot y_{i}\}$$

$$s.t.:\;\;\;\;\;\;\sum_{i=1}^{N}(\alpha_{i}^{*}-\alpha_{i})=0,$$

$$\;\;\;\;\;\;0\leq \alpha_{i}^{*}\leq \frac{C}{N},$$

$$\;\;\;\;\;\;\sum_{i=1}^{N}(\alpha_{i}+\alpha_{i}^{*})\leq C\cdot v,i=1,\ldots ,N.$$

LS-SVR: the authors of [64] define the dual problem of LS-SVR as

(15) $$Max\{g_{D_{LS-SVR}}=-\frac{1}{2}\sum_{i=1}^{N}\sum_{j=1}^{N}\left(\alpha_{i}\cdot \alpha_{j}\cdot K\left(A_{i},A_{j}\right)\right)+\sum_{i=1}^{N}\left(\alpha_{i}\cdot y_{i}\right)-\frac{N}{2C}\cdot \sum_{i=1}^{N}\alpha_{i}^{2}\}$$

$$s.t.:\;\;\;\;\;\;\sum_{i=1}^{N}\alpha_{i}=0.$$

TSVR: the authors of [34] define the dual problem of TSVR, as

(16) $$Max\{g_{D_{TSVR}}=-\frac{1}{2}\alpha^{T}H{\left(H^{T}H\right)}^{-1}H^{T}\alpha +f^{T}H{\left(H^{T}H\right)}^{-1}H^{T}\alpha -f^{T}\alpha \}$$

$$s.t.:\;\;\;\;\;\;0\leq \alpha \leq C_{1}e.$$

(17) $$Max\{g_{D_{TSVR}}=-\frac{1}{2}\gamma^{T}H{\left(H^{T}H\right)}^{-1}H^{T}\gamma -h^{T}H{\left(H^{T}H\right)}^{-1}H^{T}\gamma +h^{T}\gamma \}$$

$$s.t.:\;\;\;\;\;\;0\leq \gamma \leq C_{2}e.$$

where, $H=\left[K(A,A^{T})e\right]$.

**Figure 4:**
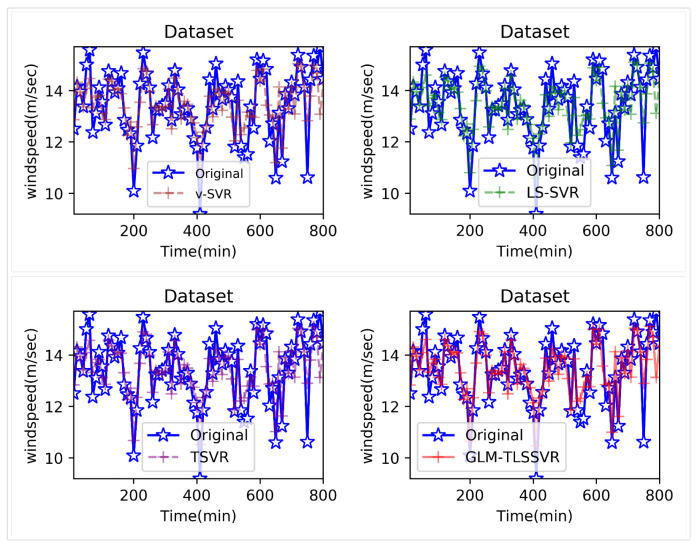
Result of four short-term wind speed forecasting models after 10 min.

**Figure 5:**
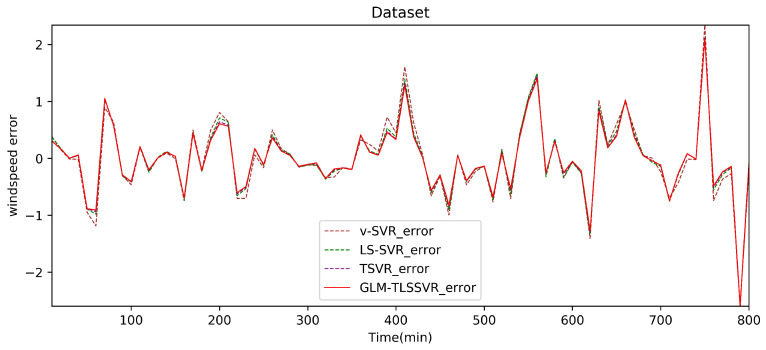
Error of four short-term wind speed forecasting models after 10 min.

**Figure 6:**
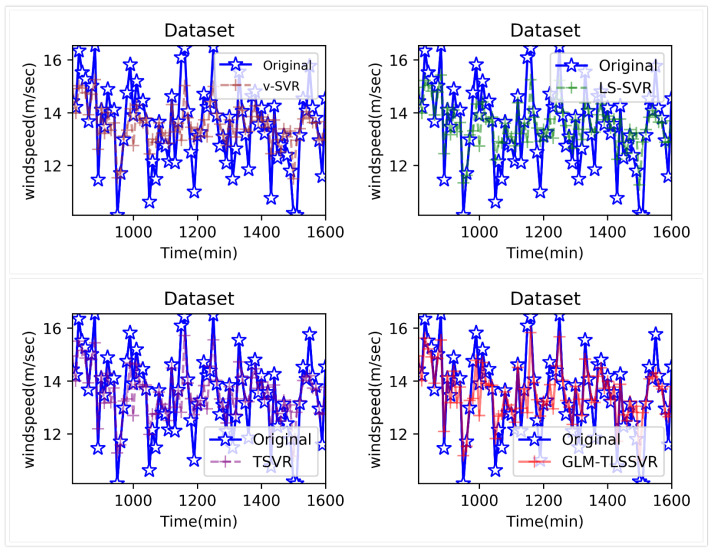
Result of four short-term wind speed forecasting models after 30 min.

**Figure 7:**
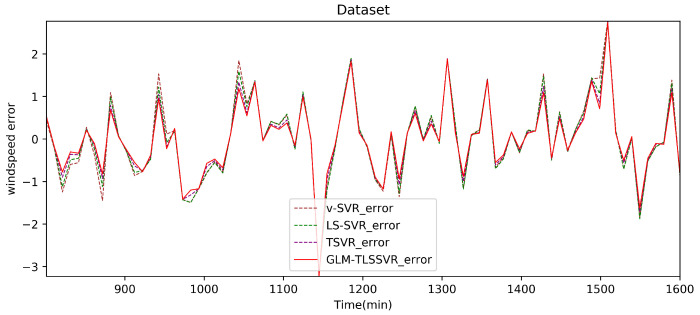
Error of four short-term wind speed forecasting models after 30 min.

**Figure 8:**
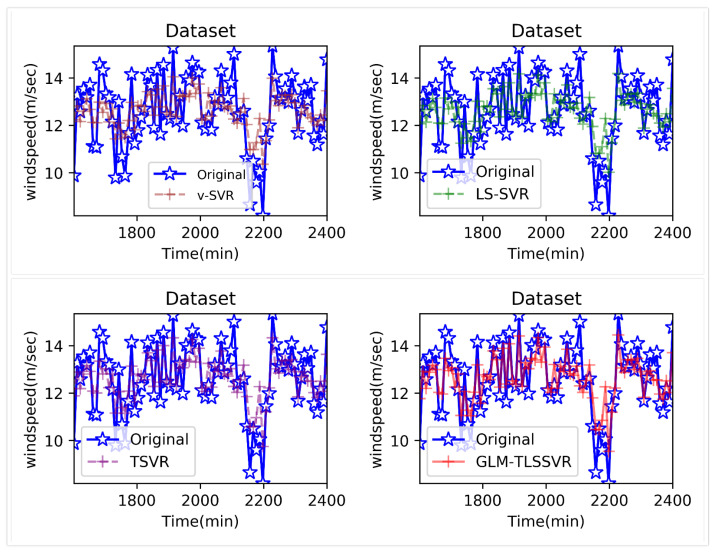
Result of four short-term wind speed forecasting models after 50 min.

**Figure 9:**
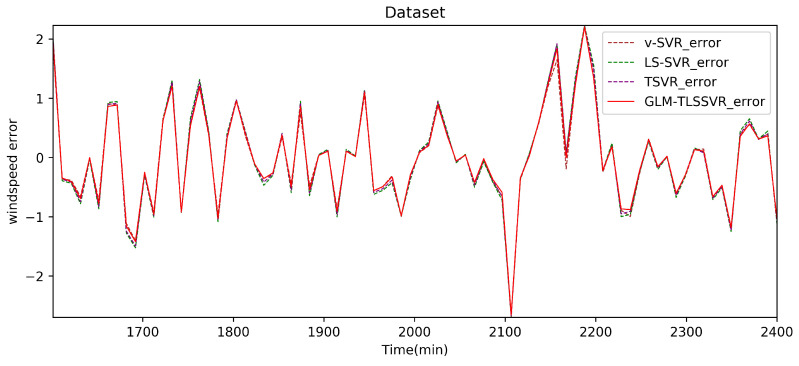
Error of four short-term wind speed forecasting models after 50 min.

In Figure 4, wind-speed forecasting-results at $A_{i}$-point of the above four models are presented after 10 min. Figure 5 shows the error statistic of wind-speed prediction using the above four models after 10 min. In Figure 6, wind-speed forecasting-results at $A_{i}$-point of the above four models are presented after 30 min. Figure 7 shows the error statistic of wind-speed prediction using the above four models after 30 min. In Figure 8, wind-speed forecasting-results at $A_{i}$-point of the above four models are presented after 50 min. Figure 9 shows the error statistic of wind-speed prediction using the above four models after 50 min. Table 2, Table 3 and Table 4 display the statistical criteria of MAE, RMSE, SSE/SST, SSR/SST, and teTime.

From Table 2, Table 3 and Table 4 and Figure 4, Figure 5, Figure 6, Figure 7, Figure 8 and Figure 9, these evaluation criteria can indicate that the error statistic of GLM-TLSSVR model is better than that of models ν-SVR, LS-SVR, and TSVR. As the forecast time interval increases from 10-min. to 30-min. and 50-min., the forecasting error of the four models increases and the relative error decreases. Therefore, in these cases, it is not so important. However, as can be seen from Table 2, Table 3 and Table 4, under all conditions of MAE, RMSE, SSE/SST, and SSR/SST, the model GLM-TLSSVR with Gaussian–Laplace mixed noise characteristics is slightly better than the other three classical ν-SVR, LS-SVR and TSVR models. In general, a lower value of MAE, RMSE, and SSE/SST reflects the consistency between the predicted values and true values, while the higher values of SSR/SST indicate that the regressor accounts for higher statistical information. Further, the performance indices indicate that GLM-TLSSVR outperforms ν-SVR, LS-SVR, and TSVR for short-term wind speed data set in terms of SSE/SST, RMSE, and MAE. The ratio of SSR/SST can estimate the goodness of fit of the predictive model and extract the maximum information from the data set. Therefore, the proposed model GLM-TLSSVR is considered to be the best regression indicator among all of the models. The SSE/SST is lower for GLM-TLSSVR when compared to other methods that imply good estimation between real values and predictive values from Table 2, Table 3 and Table 4. In addition, among all of the models, the computational cost of testing model GLM-TLSSVR is the lowest, which indicates that our proposed iterative methods are the efficient algorithm for regression, on the other hand, the reason is that this proposed model GLM-TLSSVR combines the spirit with the fast speed of LS-SVR yields a new regressor. In addition, the generalization performance of the proposed model GLM-TLSSVR is best, i.e., it owns the smallest and largest evaluation criteria, respectively, viz. RMSE, and SSR/SST from Table 2, Table 3 and Table 4; this is mainly due to the idea of twin hyperplanes.

## 6. Conclusions

Many regression techniques today assume that this model is a single noise characteristic. Wind speed prediction is complicated by its volatility and uncertainty, so it is difficult to model with a single noise distribution. This section summarizes our main work: (1) we use the Bayesian principle to derive the best empirical risk loss of G-L mixed noise characteristics; (2) the TLSSVR model of G-L mixed homoscedastic noise (GLM-TLSSVR) and G-L mixed heteroscedastic noise (GLMH-TLSSVR) for complicate noise is developed; (3) use the Lagrange function and obtain the dual problem of GLM-TLSSVR and GLMH-TLSSVR according to KKT conditions; (4) solve the GLM-TLSSVR by the ALM method, ensuring the stability and effectiveness of the algorithm; (5) use the proposed technique to predict the future short-term wind speed, calculate wind speed based on past data, and then predict wind speed at some time after 10, 30, and 50 min, respectively. Based on our results, it is observed that GLM-TLSSVR outperforms ν-SVR, LS-SVR, and TSVR for the short-term wind speed data-set, as shown in the experiment. Further, the ratio of SSR/SST can estimate the goodness of fit of the predictive model and extract the maximum information from the data set. Therefore, the proposed model GLM-TLSSVR is considered to be the best regression indicator among all of the models. A low ratio of SSE/SST implies good estimation between real values and predictive values. In addition, the computational time for all the models is evaluated and it is found that GLM-TLSSVR is the lowest, owing to its smaller sized constrained optimization. These results also bring many benefits to the industrial sector, such as better statistical analysis of the relationship between wind speed characteristics and power generation.

There are uncertainties in the data in some actual regression problems. Uncertainty, like this accident, is mainly reflected in the uncertain time of the accident, the uncertain situation of the accident, and the uncertain direction of the accident. We should study the regression algorithm of fuzzy uncertainty with mixed noise characteristics models. In addition, our work only discusses the problem of regression models with Gaussian–Laplace mixed noise characteristics. In fact, we can develop similar problems to classification learning. In a similar idea, we can still study the classification problems with Gaussian–Laplace mixed noise characteristics in the future.

## Figures and Tables

**Table 1:** Evaluation criteria for short-term wind speed prediction.

Parameter	Mathematical Expression
MAE	$\frac{1}{L}\sum_{i=1}^{N}\left|y_{i}^{*}-y_{i}\right|$
RMSE	$\sqrt{\frac{1}{L}\sum_{i=1}^{N}{\left(y_{i}^{*}-y_{i}\right)}^{2}}$
SSE	$\sum_{i=1}^{L}{\left(y_{i}^{*}-y_{i}\right)}^{2}$
SSR	$\sum_{i=1}^{L}{\left(y_{i}^{*}-\bar{y}\right)}^{2}$
SST	$\sum_{i=1}^{L}{\left(y_{i}-\bar{y}\right)}^{2}$
SSE/SST	$\sum_{i=1}^{L}{\left(y_{i}^{*}-y_{i}\right)}^{2}/{\left(y_{i}-\bar{y}\right)}^{2}$
SSR/SST	$\sum_{i=1}^{L}{\left(y_{i}^{*}-\bar{y}\right)}^{2}/{\left(y_{i}-\bar{y}\right)}^{2}$

**Table 2:** Error statistics of four short-term wind speed forecasting models after 10 min.

Model	MAE (m/s)	RMSE (m/s)	SSE/SST	SSR/SST	teTime (s)
ν-SVR	0.4797	0.6799	0.2603	0.4552	0.68
LS-SVR	0.4434	0.6366	0.2282	0.5064	0.66
TSVR	0.4182	0.6161	0.2137	0.5270	0.56
GLM-TLSSVR	0.4091	0.6069	0.2074	0.5384	0.55

**Table 3:** Error statistics of four short-term wind speed forecasting models after 30 min.

Model	MAE (m/s)	RMSE (m/s)	SSE/SST	SSR/SST	teTime (s)
ν-SVR	0.7596	1.0041	0.4378	0.2365	0.71
LS-SVR	0.7131	0.9466	0.3891	0.2932	0.68
TSVR	0.6167	0.8546	0.3171	0.3793	0.59
GLM-TLSSVR	0.5787	0.8204	0.2923	0.4197	0.57

**Table 4:** Error statistics of four short-term wind speed forecasting models after 50 min.

Model	MAE (m/s)	RMSE (m/s)	SSE/SST	SSR/SST	teTime (s)
ν-SVR	0.7781	0.9877	0.4333	0.2227	0.77
LS-SVR	0.7252	0.9202	0.3761	0.2714	0.69
TSVR	0.6566	0.8485	0.3198	0.3287	0.65
GLM-TLSSVR	0.6121	0.8005	0.2847	0.3702	0.58

## Abbreviations

The following abbreviations are used in this manuscript:

ν-SVR	ν-Support vector regression
LS-SVR	Least squares support vector regression model
TSVR	Twin support vector regression model
GLM-TLSSVR	Twin LS-SVR model of Gaussian-Laplacian mixed homoscedastic-noise
ALM	Augmented Lagrange multiplier method
